# Anti-Proliferative Effect and Phytochemical Analysis of *Cymbopogon citratus* Extract

**DOI:** 10.1155/2014/906239

**Published:** 2014-03-27

**Authors:** Mohammed F. Halabi, Bassem Y. Sheikh

**Affiliations:** ^1^Department of Biomedical Sciences, Faculty of Medicine, University of Malaya, 50603 Kuala Lumpur, Malaysia; ^2^Al-Moalim Mohamed Awad Center for Scientific Miracles of Prophetic Medicine, College of Medicine, Taibah University, 3001 Madinah, Saudi Arabia; ^3^Department of Neurosurgery, College of Medicine, Taibah University, 3001 Madinah, Saudi Arabia; ^4^Faculty of Medicine, P.O. Box 30001, Al-Madinah Al-Munawarah, Saudi Arabia

## Abstract

The antiproliferative and antioxidant potential of *Cymbopogon citratus* (Lemon grass) extracts were investigated. The extracts were isolated by solvent maceration method and thereafter subjected to antiproliferative activity test on five different cancer cells: human colon carcinoma (HCT-116), breast carcinoma (MCF-7 and MDA-MB 231), ovarian carcinoma (SKOV-3 and COAV), and a normal liver cell line (WRL 68). The cell viability was determined using MTT assay. The DPPH radical scavenging assay revealed a concentration dependent trend. A maximum percentage inhibition of 45% and an IC50 of 278 **μ**g/mL were observed when aqueous extract was evaluated. In contrast, 48.3% and IC50 of 258.9 **μ**g/mL were observed when 50% ethanolic extract was evaluated. Both extracts at concentration of 50 to 800 **μ**g/mL showed appreciative metal chelating activity with IC50 value of 172.2 ± 31 **μ**g/mL to 456.5 ± 30 **μ**g/mL. Depending on extraction solvent content, extract obtained from 50% ethanolic solvent proved to be more potent on breast cancer MCF-7 cell line (IC50 = 68 **μ**g/mL). On the other hand, 90% ethanolic extract showed a moderate potency on the ovarian cancer (COAV) and MCF-7 cells having an IC50 of 104.6 **μ**g/mL each. These results suggested antiproliferative efficacy of *C. citratus* ethanolic extract against human cancer cell lines.

## 1. Introduction

Cancer is among the leading causes of mortality among human population of all ages. In fact, it is responsible for 7.6 million deaths in 2008 [1]. It has been projected that the cancer mortality rate will extend to about 30.1 million by 2030 [1]. Current therapeutic interventions mostly involve malign surgery, radiotherapy, and chemotherapy, and at times the therapeutic efficiency is very low. This incurs the current increase in research on mild alternative cancer therapy. Bioactive phytochemicals exhibiting the ability to inhibit cancer cytogenesis by suppressing the tumor initiation, promotion, and progression are being considered as potential biocompatible anticancer agents. In this regard, the antiproliferative activity of several phytochemical extracts was reported [2–4].

Among the medicinal plants used,* Cymbopogon citratus* (lemon grass) is prominent and commonly explored in folk alternative medicine for the treatment of diverse ailments. Although, several bioactive compounds were reported to be isolated from* C. citratus*; among them is the acyclic monoterpene aldehydes described as citral that comprises of isomeric geranial and neral. Citral was reported to be the major bioactive component that incurs most of the plant's bio efficacy [5]. Popularly, the aqueous infusion of this plant is called “abafado” by Portuguese, and was said to have bioactive efficacy against nervous and gastrointestinal disturbances when administered orally [6]. In addition, it is also reported to be a potent free radical scavenger of reactive oxygen species [7]. Furthermore, citral was shown to possess activities like anti mutagenicity [8], antiproliferative effect against* Trypanosoma cruzi *[9], and antinociceptive and[10], antiparasitic effects against leishmaniasis [11, 12].

The efficient potency of* C. citratus* on free radical scavenging and antioxidation ability led us to evaluate the effect of its aqueous ethanolic extracts on proliferation and cell growth of several human cancer cell lines such as those of breast cancer [MDA-MB 231 and MCF-7], ovarian cancer [SKOV-3 and COAV], and colon cancer [HCT-116]. In addition, the phytochemical content analysis of the extracts is also reported.

## 2. Materials and Methods

### 2.1. Plant Materials, Phytochemicals Extraction and GCMS Analysis

*C. citratus* leaves were identified and obtained by MABLEAJAZ chair for Scientific Research in Prophetic Medicine, Faculty of Medicine, Taibah University, Saudi Arabia (Specimen Voucher Number: TU/JX03/SP2765). The leaves were cleaned, dried under shade for 7 days then grounded, weighed, homogenized in water (Ew), 50% ethanol (E50), and 90% ethanol (E90) at a ratio of 1 : 10 of plant powder to solvent, and left to macerate for 5 days at ambient temperature (25 ± 1°C) with occasional shaking and stirring. The mixture was then filtered and the resulting liquid was concentrated under reduced pressure at 40°C in an EYELA rotary evaporator yielding a dark brown to green extracts. The concentrated extracts were then kept in* vacuo* at 45°C for 3 days to evaporate the residual solvents resulting in the respective dried crude extract of either Ew, E50, or E90, respectively. Extracts were then dissolved in 0.5% DMSO before being used in the cell cultures at concentrations of 3, 6, 12, 25, 50, and 200 µg/mL.

Another portion of the extract (1 mg) was dissolved in 1 mL methylene chloride in a screw capped test tube. To this mixture, 1 mL of acidified methanol (methanol containing 15% H_2_SO_4_) was added, then tightly capped and incubated at 100°C for 2 hours. At the end of heating, the reaction mixture was allowed to cool down to room temperature, followed by addition of 1 mL of deionized water and vortex to induce phase separation, and to stand for a minute. Using Pasteur pipette, about 1 mL of the organic phase was carefully withdrawn into GC vials for GCMS phytochemical content analysis.

The GCMS analysis was conducted on Agilent 7000B triple quadrupole GCMSMS machine carrying triple axis detector and Agilent HP-5 MS separation column that has been impregnated with 5% phenyl methyl silox (30 m long × 0.25 mm internal diameter × 0.25 µm film thickness). A sample (1 µL) was automatically injected into the machine at a split ratio of 1 : 20. The injection temperature was set at 280°C. The oven ramping temperature profile was as follows: 40°C for 2 min then increased to 140°C at 3°C min^−1^, held at 140°C for 2 min then increased to 250°C at 10°C min^−1^, and then held at 250°C for 5 min. Helium was used as the carrier gas at a flow rate of 14 mL min^−1^. Mass spectra were acquired at 1250 scan speed using electron impact energy of 70 eV at 230°C ion-source temperature and 250°C interface temperature.Corresponding spectrum for each chromatogram peak was compared with deposited spectra in NIST database for compound identification.

### 2.2. DPPH Assay

Radical scavenging activities of the extracts were determined by a spectrophotometric assay using alcoholic solution of 1,1-diphenyl-2-picrylhydrazyl (DPPH) as reported somewhere else [7]. Briefly, different concentrations of extracts-DMSO solutions (15.6–250 µg/mL) are added to a solution of DPPH (200 mM) in absolute ethanol and incubated in dark for 30 min at room temperature (25 ± 1°C). The change in chrometric status of DPPH from purple to yellow upon reduction was measured spectrophotometrically at 517 nm for each sample after incubation against a control solution of DPPH and DMSO alone. Ascorbic acid was used as standard control. The free radical scavenging activities of the extracts were calculated as a percentage of radical reduction in (1). All experiments were performed in triplicates, and the IC_50_ values were determined from a calibration curve for each extract:

(1) Radical  scavenging  activity  % =  (1−(Abs517  controlAbs517  sample  or  standared  control))×100.

### 2.3. Metal Chelating Assay

The metal chelating abilities of the extracts were determined according to the method of Wang et al. [35] with minor modifications. The stock solutions of the extracts (100 µL of 5 mg/mL) were mixed with 135 µL of distilled water and 5 µL of 2 mM FeCl_2_ in a microplate. The reaction was initiated by the addition of 10 µL of 5 mM ferrozine. Thereafter solutions were mixed and allowed to stand for 10 min at room temperature (25 ± 1°C). After incubation, the absorbance was measured at 562 nm with a microplate reader (GF-M3000, UNICOM-OPTICS). Distilled water (100 µL) instead of sample solution was used as a control. Distilled water (10 µL) in place of ferrozine solution was used as a blank, which is used for error correction because of unequal colour of the sample solutions. EDTA-Na_2_ was used as reference standard. All measurements were performed in triplicate, and the ferrous ion-chelating ability was calculated according to (2):

(2) Metal  chelating  ability%=([(Abs562  control−(Abs562  sample−Abs562  blank))]Abs562  control) ×100.

### 2.4. FRAP Assay

The ferric reducing antioxidant powers (FRAP) of the extracts were assayed according to the previously described method [36] with slight modification. In brief, the FRAP reagent was prepared by adding 300 mM acetate buffer (3.1 mg sodium acetate/mL, pH 3.6) to 10 mM 2,4,6-tripyridyl-S-triazine (TPTZ) solution and 20 mM FeCl_3_·H_2_O (5.4 mg/mL). A portion of TPTZ reagent (290 *μ*L) was added to each well of 96-well titre plate in triplicate, and aliquot sample (10 *μ*L) of 1 mg/mL of prepared* C. citratus* extracts was used to read the absorbance at 593 nm in ELISA reader (Shimadzu, Japan) after every 4 min for 2 h.

### 2.5. Nitric Oxide Assay

Nitric oxide (NO) scavenging activity of the extracts was determined using Griess reaction. According to this reaction, when sodium nitro-prusside is used in aqueous solution at physiological pH, it generated NO^•^ radicals that react with oxygen to produce nitrite ions. The produced ions are then quantified via spectrophotometric analysis as reported by Nagmoti et. al. [37]. In brief, 1 µL of 10 mM sodium nitro-prusside was mixed with 1 mL of test extracts or curcumin (as a reference control) at various concentrations (400–1600 µg/mL) dissolved in methanol and a control without test extracts, but only with an equivalent amount of methanol. The mixture was then incubated for 30 min at room temperature (25°C). After 30 min of incubation, 1 mL of Griess reagent (1% sulphanilamide, 2% phosphoric acid, and 0.1% naphthyl ethylenediamine dihydrochloride) was added to 1 mL of the incubated solution and vortex. The absorbance of the pink coloration during the diazotization of the nitrite with sulphanilamide and the subsequent coupling with naphthyl ethylenediamine dihydrochloride was measured at 546 nm. All the tests were performed in triplicate. Percentage inhibition was calculated using (3):

(3) NO  scavenging  % =([Abs546  control  −  Abs546  sample]Abs546  control)×100.

### 2.6. Evaluation of* C. citratus* Cytotoxicity by MTT Assay

Three different extracts were prepared for studying the antiproliferative effect of* C. citratus* extracts against the human cancer cell lines in reference to normal liver cell line WRL 68, these were Ew, E50, and E90 representing aqueous and 50% and 90% ethanol, respectively. Both the normal cell line (WRL 68) and the human cancer cell lines were used (breast cancer [MDA-MB 231 and MCF-7], ovarian cancer [SKOV-3 and COAV], and colon cancer cell lines [HCT-116]) were obtained from American Type Culture Collection and supplied by Department of Molecular Medicine, University of Malaya. Extract's antiproliferative effect by MTT (3-(4,5-dimethylthiazol-2-yl)-2,5-diphenyltetrazolium bromide) assay was evaluated according to modified protocol [38]. Briefly, cell lines were cultured in RPMI-1640 growth medium and supplemented with* C. citratus* extracts at different concentrations (3–200 µg/ml), 10% (v/v) sterile fetal bovine serum (FBS, PAA Lab, Austria), 100 mg/mL streptomycin, 100 U/mL penicillin (PAA Lab, Austria) and 50 mg/mL fungizone (Sigma Aldrich). Cultures were incubated in 5% CO_2_ incubator at 37°C in a humidified atmosphere. The cells were harvested by detaching the cells from the culture flask using trypsin after the flask get confluent enough with the cells. The harvested cells were then aseptically introduced into 50 mL sterile falcon tube and washed with physiological buffer (pH 7.2) under spinning at 1200 rpm for 10 minutes. The supernatant was discarded, and the cells pellets were mixed with 1 mL of sterile media to form a cell suspension. The viable cells count was determined using trypan blue assay. About 10 µL of the cell suspension was mixed with 10 µL of trypan blue, and aliquot sample (10 µL) of this mixture was used to count the cells in Neo Bar chambers.

The Harvested cells were then seeded into 96-well culture plates at 5000 cells/well and allowed to adhere overnight. Both the water and alcoholic extracts of the* C. citratus* were dissolved in 5% dimethyl sulphoxide (DMSO) with final concentration of 0.5% and diluted to different concentration spanning from 3–200 µg/mL. Blank 5% DMSO was used as a control. Cells were incubated with the samples (three wells on a plate for each concentration) from 48 to 72 h. Thereafter, 10 µL of MTT (5 mg/mL) (Sigma) was added to each well and the plates were incubated at 37°C for 4 h. The media was then gently aspirated, and about 200 mL of DMSO was added to dissolve the formazan crystals. The amount of formazan product was measured spectrophotometrically at 570 nm using a microplate reader (GF-M3000). The percentage cell viability was calculated according to (4):

(4) $$\begin{array}{c} \text{Cell}\;\text{viability}\;\%=\left(\frac{{\text{Abs}}_{570}\;\text{treated}}{{\text{Abs}}_{570}\;\text{untreated}}\right)\times 100. \end{array}$$

### 2.7. Statistical Analysis

The obtained experimental data were evaluated statistically as mean ± S.E.M. (standard error mean). The statistical differences between the groups were determined based on 95% confidence intervals using one-way ANOVA in SPSS program (SPSS Inc., USA). All obtained data were analyzed using Analysis of Variance (ANOVA). A probability value of *P* < 0.05 was considered as significant between the measurements of the two compared groups.

## 3. Results and Discussion

### 3.1. Analyses of Phytochemical Extracts, Radical Scavenging, and Antioxidant Efficacy

**Figure 1:**
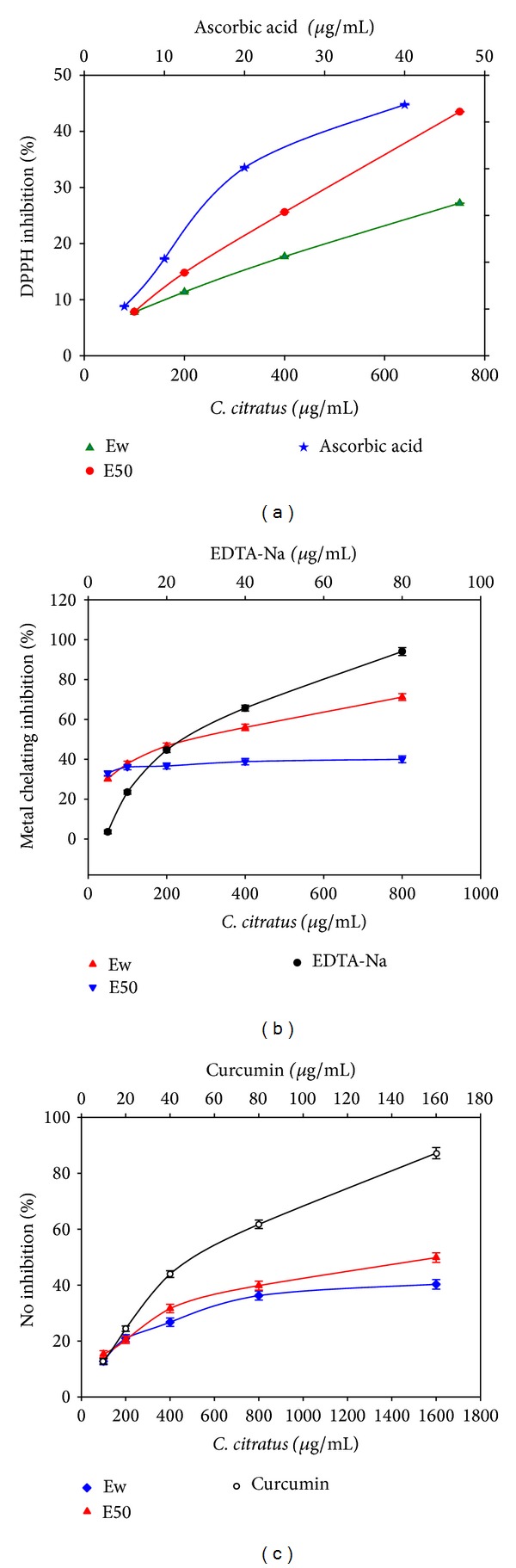
Antioxidant and radical scavenging property of* C. citratus *aqueous (Ew) and 50% ethanolic (E50) extracts. (a) DPPH radical scavenging activity. (b) Metal chelating capacity. (c) NO inhibition activity. All values are expressed as the means ± S.E.M. Statistical difference is significant at the *P* < 0.05 level.

In this research, maceration using aqueous-solvent extraction of lemon grass yielded 5.4 g/100 g on dry weight basis. Based on GCMS qualitative analysis, eighteen (18) phytochemicals compounds from the extracts were identified (Table 1). The analysis revealed that the constituents of lemon grass extracts mostly belong to monoterpene, sesquiterpene, and phenolic acids. This phytochemical composition analysis was found to be in accord with previously reported studies [39, 40]. The antioxidants potential of* C. citratus* leaves extracts on the DPPH radical scavenging were determined by their hydrogen donating ability. Evaluating the DPPH radical scavenging activity of both Ew and E50 extracts against ascorbic acid as a standard control (Figure 1(a)), it is observed that at lower concentrations (100–200 µg/mL) both extracts revealed almost similar radical scavenging activity (Figure 1(a)). However, as the concentration increases, the Ew extract seems to have a logarithmic increase in percentage inhibition over the concentration range, achieving a maximum inhibition of about 45% and an IC_50_ value of about 278 as shown in Figure 1(a). On the other hand, increasing the concentration of E50 extract to 750 µg/mL revealed an increase in percentage inhibition. Beyond this value, the percentage inhibition appeared to approach plateau with the increment in the extract's concentration attaining a maximum percent inhibition of 48.3% with corresponding IC_50_ of 258.90 (Figure 1(a)). This could be due to the difference in the extracted terpenes content. When compared to the ascorbic acid, it takes about 1500 µg/mL of the extract to achieve the percentage inhibition of the standard control at a concentration about 20 µg/mL with corresponding IC_50_ of 6.21 µg/mL. These observations were found to be in agreement with similar observations on a higher IC_50_ values for* C. citratus* extracts [41–43].

The antioxidant results presented so far indicated that the phytochemical extracts of* C. citratus* leaves had both proton and electron donating abilities of primary antioxidant efficacy. However, it has been reported that secondary antioxidants serve as effective ligands in chelating metal ions, thereby suppressing the formation of hydroxyl radicals by Fenton's reaction [44]. Interestingly, in this assay, both the Ew and E50 extracts at concentrations of 50 to 800 µg/mL showed appreciative metal chelating activity (Figure 1(b)). Among the extracts studied, the highest activity was observed in Ew with corresponding metal chelating inhibition of 71.2% and an IC_50_ value of 172.2 ± 31 µg/mL, while the E50 extract showed the chelating inhibition of 40% and a corresponding IC_50_ value of 456.5 ± 30 µg/mL. In contrast to the standard control (EDTA-Na), the observed results were found to equal the standard's chelating performance of about 40 µg/mL (Figure 1(b)).

The ferric ion reducing capacity (FRAP) showed both the extracts to possess almost similar activity. The assay activity trend revealed an increase in value from 0.21 Fe^2+^/g in the Ew extract to 0.3 Fe^2+^/g in the E50 extract, signifying the effectiveness of the E50 extract as compared to the Ew extract. Runnie et al. [45] reported similar observation on the FRAP activity of* C. citratus* leaf extract. Within the animal cells, it is a fact that most inflammatory responses were associated with nitric oxide [46]. In this study, both the Ew and E50 extracts were checked for their inhibitory effect against nitric oxide production (Figure 1(c)). Among the analyzed samples and reference to the standard curcumin (IC_50_ = 10.8 ± 1.2 µg/mL), the E50 extract showed the highest nitric oxide inhibitory activity of 49.3% (IC_50_ 462.9 ± 29 µg/mL) as compared to the Ew extract of 40.1% (432.4 ± 24 µg/mL). In fact, the extracts were found to have a nitric oxide scavenging activity equivalent to about 40 µg/mL of the standard curcumin (Figure 1(c)).

### 3.2. Antiproliferative Effect of* C. citratus* Extract on Different Cancer Cell Lines

**Figure 2:**
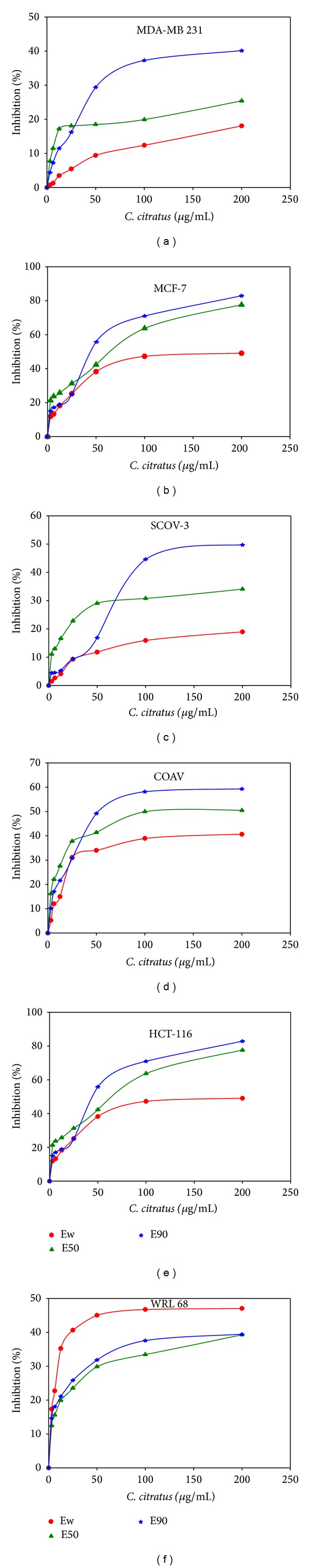
Antiproliferative efficacies of* C. citratus *extracts by MTT assay on (a) MDA-MB 231 (b) MCF-7, (c) SCOV-3, (d) COAV, (e) HCT-116, and (f) WRL 68 cell lines. Ew, E50, and E90 represent the aqueous, 50% and 90% ethanolic extracts, respectively. All values are expressed as the means ± S.E.M. Statistical difference is significant at the *P* < 0.05 level.

The effects of extracts concentrations on the viability and growth of tumor cell lines in reference to the normal cell line have been observed (Figures 2(a)–2(f)). Generally, when the extracts treatments in cancer cell lines (Figures 2(a)–2(e)) were compared with that of the normal cell line WRL 68 (Figure 2(f)), the antiproliferative effect of the extract could be observed to be exerted more on the cancer cell lines, the extract was observed to cause an inhibition of less than 50% even at higher dosage (200 µg/mL). On the other hand, using the extract even at lower concentrations <50 µg/mL caused marked inhibition in the cell growth. At the lower concentrations (i.e. <50 µg/mL), it is observed that E50 extract has the highest inhibition activity. However, as the treatment concentration increases beyond 50 µg/mL, E90 showed the highest efficacy (Figures 2(a)–2(e)). Among the breast cancer cell lines tested, E90 showed lower inhibition in MDA-MB 231 cell line with corresponding 40% inhibition (Figure 2(a)) and IC_50_ >200 µg/mL as compared to observed inhibition activity on MCF-7 cell line (Figure 2(b)), which showed percent inhibition of 82.9% and IC_50_ of 104.6 µg/mL. In comparison to E90 extract, E50 was observed to be more potent on MCF-7 cell line (IC_50_ = 68 µg/mL) than on MDA-MB 231 cell line (IC_50_ ≥ 200 µg/mL). It has been reported that the antiproliferative activities of phytochemical extracts could be due to their influence on incurring increase expression of kinase and their activities of positive G1/S and G2/M regulators with simultaneous expression of p21 in presence of high level of p27 and p53 [4]. These effects caused a blockade in cell cycle, thus inducing the apoptosis in MCF-7 cells.

Evaluating the extract's antiproliferative effect on SCOV-3 (Figure 2(c)) and COAV (Figure 2(d)) cell lines, increasing the E90 concentration to 100 µg/mL resulted in a logarithmic increase in percentage inhibition with an observed IC_50_ of 200 and 104.6 µg/mL in SCOV-3 and COAV, respectively. Thereafter, increasing the concentration above 100 µg/mL resulted in minimal increase in the percentage inhibition achieving a maximum inhibition of about 50% and 59% in both SCOV-3 and COAV, respectively. This stabilization in the percentage inhibition at higher concentration could be attributed to the major death of the cell population. Similar observation has been reported by Konrad et al. [47], who observed a reduction in growth inhibition with increasing* Urtica dioica* root extract concentration on human prostate cancer (LNCaP) cell line. Analyzing the extract's efficacy on colon cancer cell line HCT-116 (Figure 2(e)), revealed a different trend, since the percentage inhibition appeared to continue to increase with increasing concentration up to 200 µg/mL resulting in maximum inhibition of about 83% and IC_50_ > 200 µg/mL. According to Dudai et al. [48] the mechanism of the inhibition is mostly accompanied by DNA fragmentation and caspase-3 catalytic activity induction. Although at higher ethanolic content extraction there is an appreciative inhibitory activity, the extract performance was found to be lower than that of aqueous extracts. In this extract all samples tested were found to have an IC_50_ > 200 µg/mL. In general, the results presented here on cell growth inhibition by* C. citratus* extract are further supported in the light of the antiproliferative effects of the plant's phytochemical constituents that have been published before as shown in Table 1.

## 4. Conclusions

The oxidative radical scavenging and chemo-preventive efficacy of* C. citratus* extracts were evaluated. Ew and E50 extracts have shown DPPH antioxidant activities. In addition, the extracts were found to show an appreciative iron chelating and nitric oxide scavenging activities. On evaluating the antiproliferative effect, five (5) human cancer cell lines were compared in this analysis. The E50 extract proved to be more potent on breast cancer MCF-7 cell line. On the other hand, E90 extract showed a moderate potency on the ovarian cancer (COAV) and MCF-7 cell lines. Compared to extracts from higher ethanolic content, aqueous extracts were observed to show lower inhibitory activity with a generalized IC_50_ > 200 µg/mL in all samples tested. In general, the observed efficacy could probably be due to the phytochemical constituents of this plant.

## Figures and Tables

**Table 1:** Phytochemical composition of aqueous extract of *Cymbopogon citratus*.

Number	Compound	Extract		Reported application	Reference
		Ew	E50		
		%	%		
1	*Hydroquinone *	0.8	ND	Treatment of melasma	[13]
2	*Nerolidol *	24	ND	Anticancer	[14]
3	*β-Elemene *	42	ND	Anticancer	[15]
4	*β-Eudesmol *	12	ND	Anticancer	[16]
5	*Myrtenal *	4	0.6	(i) Inhibits Alzheimer's acetylcholinesterase (ii) Anticancer (iii) Insect repellent	[17] [18] [19]
6	*Piperitone *	2.1	4.5	Antimicrobial	[20] [21]
7	*Nonadiyne *	ND	0.6	Inhibits the release of endogenous nitric oxide	[22]
8	**α*-Cubebene *	ND	0.5	Cytotoxic and antimicrobial	[23]
9	**α*-Copaene *	ND	0.3	Antidiabetic	[24]
10	*L-calamenene *	ND	0.2	Anticancer	[25]
11	*Elemol *	ND	41	Antimosquitoes	[26]
12	*Humulene *	ND	4	Anti-inflammatory and inhibits the generation of tumor necrosis factor-*α* (TNF-*α*) and interleukin-1 *β* (IL1*β*)	[27]
13	*Caryophyllene *	ND	0.9	Antiulcerogenic and anti-inflammatory	[28]
14	*Cubenol *	ND	2	Cytotoxic effect	[29]
15	*Cubebol *	ND	4.7	Mosquito larvicidal activity	[30]
16	*Carvone *	0.7	2	Sprout suppression and antifungal activity in potato	[31]
17	*β-Eudesmol *	ND	45	Antimutagenic Antiangiogenic	[32] [33]
18	*Nonanenitrile *	ND	0.9	Antipancreatitis and antiulcerogenic	[34]

*ND: not detected.
